# Effect of intravenous palonosetron on hypotension induced by spinal anesthesia for cesarean section: A randomized controlled trial

**DOI:** 10.1371/journal.pone.0305913

**Published:** 2024-06-25

**Authors:** Min Kyoung Kim, Injeong Kim, Hyun Kang, Wongook Wi, Yong Hee Park, Yong Hun Jung, Young Cheol Woo, Chong Wha Baek

**Affiliations:** 1 Department of Anesthesiology and Pain Medicine, College of Medicine, Chung-Ang University, Seoul, South Korea; 2 Department of Anesthesiology and Pain Medicine, Chung-Ang University Gwangmyeong Hospital, Gyeonggi-do, South Korea; 3 Department of Anesthesiology and Pain Medicine, Chung-Ang University Hospital, Seoul, South Korea; Mansoura University Hospital, EGYPT

## Abstract

**Background:**

The aim of this study was to evaluate the impact of intravenous palonosetron compared to ondansetron on hypotension induced by spinal anesthesia in women undergoing cesarean section.

**Methods:**

Fifty-four women scheduled for elective cesarean section were, randomly allocated to ondansetron group (n = 27) or palonosetron group (n = 27). Ten minutes prior to the administration of spinal anesthesia, participants received an intravenous injection of either ondansetron or palonosetron. A prophylactic phenylephrine infusion was initiated immediately following the intrathecal administration of bupivacaine and fentanyl. The infusion rate was titrated to maintain adequate blood pressure until the time of fetal delivery. The primary outcome was total dose of phenylephrine administered. The secondary outcomes were nausea or vomiting, the need for rescue antiemetics, hypotension, bradycardia, and shivering. Complete response rate, defined as the absence of postoperative nausea and vomiting and no need for additional antiemetics, were assessed for up to 24 hours post-surgery.

**Results:**

No significant differences were observed in the total dose of phenylephrine used between the ondansetron and palonosetron groups (387.5 μg [interquartile range, 291.3–507.8 μg versus 428.0 μg [interquartile range, 305.0–507.0 μg], *P =* 0.42). Complete response rates also showed no significant differences between the groups both within two hours post-spinal anesthesia (88.9% in the ondansetron group versus 100% in the palonosetron group; *P =* 0.24) and at 24 hours post-surgery (81.5% in the ondansetron group versus 88.8% in the palonosetron group; *P =* 0.7). In addition, there was no difference in other secondary outcomes.

**Conclusion:**

Prophylactic administration of palonosetron did not demonstrate a superior effect over ondansetron in mitigating hemodynamic changes or reducing phenylephrine requirements in patients undergoing spinal anesthesia with bupivacaine and fentanyl for cesarean section.

## Introduction

Cesarean section is one of the most frequently performed surgical procedures worldwide [1], and neuraxial anesthesia is generally preferred method in the absence of contraindications. Neuraxial anesthesia offers multiple benefits over general anesthesia, including reduced postoperative pain, lower risk of hemorrhage, and improved fetal outcomes [2]. Nevertheless, hypotension is a common side effect, especially in spinal anesthesia, and numerous studies has focused for achieving hemodynamic stability during spinal anesthesia.

Phenylephrine is the recommended vasopressor for countering spinal anesthesia-induced hypotension. However, its administration can result in a dose-dependent reduction in heart rate and cardiac output [3]. Consequently, research has been conducted on adjuvant strategies to minimize the required dose of phenylephrine. 5-hydroxytryptamine subtype 3 (5-HT_3_) receptor antagonists have been shown to mitigate not only postoperative nausea and vomiting (PONV) but also the incidence of hypotension and vasopressor requirements associated with spinal anesthesia [4–7]. Among 5-HT_3_ receptor antagonists, second-generation antagonists like palonosetron are increasingly favored for PONV prevention due to their enhanced potency and longer duration of action compared to first-generation antagonists like ondansetron. A study by Shin et al. has further indicated that second-generation 5-HT_3_ receptor antagonists are more effective in preventing spinal anesthesia-induced hypotension than first-generation antagonists [8].

Current guidelines recommend administering ondansetron before the end of surgery to prevent PONV, while palonosetron is recommended at the beginning of surgery [9]. To mitigate spinal anesthesia-induced hypotension, a 5-HT_3_ receptor antagonist should ideally be administered before spinal anesthesia begins. However, there is a lack of clinical trials comparing the efficacy of palonosetron and ondansetron in this specific context. We hypothesized that preoperative palonosetron administration would be superior to ondansetron in decreasing the phenylephrine requirement during spinal anesthesia for cesarean section.

## Methods

This randomized and double-blinded study was approved by the Institutional Review Board of Chung-Ang University Hospital (2107-017-470). The study protocol was registered with the Clinical Research Information Service (CRIS: https://cris.nih.go.kr; registration number: KCT0006966). The research was conducted following the 2013 Declaration of Helsinki, and written informed consent was obtained from all participants. This manuscript was written in accordance with the Consolidated Standards of Reporting Trials statement [10].

We included 54 participants with normal singleton pregnancies and an American Society of Anesthesiologists physical status of Ⅱ who were scheduled for elective cesarean section under spinal anesthesia at Chung-Ang University Hospital between February 8, 2022, and September 30, 2022. Exclusion criteria were cardiovascular, cerebrovascular, renal, hepatic, or hematologic abnormalities, body weight less than 45 kg or greater than 100 kg, emergency cesarean section, and pregnancy-related complications such as gestational hypertension, diabetes mellitus, pre-eclampsia, eclampsia, and placenta previa. Participants who declined spinal anesthesia or had allergies to study drugs were also excluded.

Participants were randomized into one of two groups (ondansetron group, n = 27; palonosetron group, n = 27) through a pre-generated random table. Block randomization was employed with a block size of four, using a computer-generated random number list created by investigator MKK. The study drugs were prepared by investigator CWB in accordance with the group assignment, packaged in opaque envelope labeled solely with the subject number, handed over to investigators IJK or YHP responsible for administering spinal anesthesia (ondansetron group: ondansetron 4 mg/2 mL, palonosetron group: palonosetron 0.075 mg /1.5 mL mixed with 0.5 mL of normal saline). Investigators MKK and CWB, who were involved in group randomization, assignment, and drug preparation, were not part of any other steps of the study. Blinding was rigorously maintained in the randomization and group allocation process, except for these two investigators.

In the operating room, the subject was equipped with noninvasive blood pressure monitoring, electrocardiography, pulse oximetry, and a simple oxygen mask set to a flow rate of 5 L/min. Ten minutes prior to spinal anesthesia, the study drug, which was prepared in an opaque envelope was administered. A balanced crystalloid solution preload of 5 mL/kg was also infused over 20 minutes before induction of spinal anesthesia. A resting period of 5 minutes was observed before an anesthesiologist, uninvolved in other aspects of the study, determined the baseline blood pressure and heart rate by averaging three consecutive measurements taken at 3-minute intervals. For the spinal anesthesia procedure, the anesthesiologist used a Quincke needle to access either the L3-4 or L4-5 intervertebral space while the patient was in the left lateral position. Following confirmation of clear cerebrospinal fluid flow, a solution containing 8–10 mg of 0.5% hyperbaric bupivacaine (Marcaine Spinal 0.5% Heavy; AstraZeneca) with 10 μg of fentanyl was injected intrathecally. Subsequently, the patient was repositioned with a 15° left-tilt using a wedge, which was maintained until the surgical procedure was complete. Blood pressure and heart rate were monitored at 1-minute intervals until the fetus was delivered and thereafter at 5-minute intervals until the end of the surgery.

An infusion of phenylephrine commenced immediately after the intrathecal injection of bupivacaine at a rate of 0.24 μg/kg/min [11]. If the blood pressure falls below 80% of the baseline, indicating hypotension, a bolus of 50 μg of phenylephrine was administered. Conversely, if blood pressure exceeded 120% of the baseline, the infusion was discontinued [7]. If the blood pressure dropped below 120% of the baseline following discontinuation, the infusion was resumed. In cases where bradycardia (heart rate <55 beats/min) was observed in conjunction with hypotension, 0.5 mg of atropine was administered. In cases of nausea or vomiting, 10 mg of metoclopramide was administered as a rescue antiemetic. Nausea and vomiting were evaluated and reported until 24 hours postoperatively.

### Primary outcome

The primary outcome was the total dose of phenylephrine administered until the time of fetal delivery. To ascertain this value, we meticulously documented both the continuous infusion and bolus doses of phenylephrine.

### Secondary outcomes

The secondary outcomes were the incidence of nausea or vomiting, the requirement for rescue antiemetics, hypotension, bradycardia, and shivering. Complete response (CR) was defined as the absence of nausea and vomiting, without the need for additional antiemetic intervention within the specified time intervals. CR was assessed at 2 hours and 24 hours post-surgery. Occurrences meeting this criterion were recorded based on investigative documentation.

### Other outcomes

For safety outcomes, we conducted a comprehensive evaluation of any adverse effects associated with the utilization of 5-HT_3_ antagonists, such as headache and arrhythmia, to assess the implication of their administration. Neonatal outcomes were assessed by the Apgar score at 1 and 5 minutes post-delivery. The evaluation of all endpoints, including primary and secondary outcomes, was performed by assessors who were blinded to the group assignments.

### Sample size calculation

The primary outcome for this study was the total dose of phenylephrine administered. Data from a previous study indicated that the mean dose of phenylephrine in the ondansetron group was 316.5 ± 25.9 μg [7]. Existing literature suggests that the second-generation 5-HT_3_ antagonists yield an approximate 7% less reduction in systolic blood pressure when compared to first-generation antagonists prior to and following spinal anesthesia [8]. Therefore, we hypothesized that, by setting the minimum clinically important difference at 7% in terms of micrograms for the total phenylephrine dose, the total phenylephrine consumption in the palonosetron group would decrease by an estimated 7% compared to the ondansetron group. Utilizing an α error of 5% and a β error of 20%, the requisite sample size for each group was determined to be 24 participants. To account for potential attrition, we included 27 subjects per group, incorporating a 10% dropout rate. The sample size was calculated using t-test with PASS software, version 11 (NCSS, Kaysville, UT, USA).

### Statistical analysis

We used an intention to treat strategy; all patients were allocated to randomized group regardless of whether or not they actually received it, and included in the analysis irrespective of whether they completed the study.

For continuous variables, the distribution of the data was first evaluated for normality using the Shapiro-Wilk test. Normally distributed data are presented as the mean ± standard deviation (SD) and the groups were compared with Student’s t-test. Non-normally distributed data are expressed as the median (interquartile range) and these data were analyzed using Mann-Whitney U test.

Serially checked variables, systolic blood pressure (SBP) and heart rate (HR), met the criteria for normal distribution. Mauchly’s sphericity test indicated that the assumption of sphericity was not violated in SBP (P = 0.17). Therefore, repeated measures ANOVA (RM-ANOVA) was used to analyze between-group differences for SBPs, incorporating a within-subjects factor of time points (Base, 5 min, 10 min, 15 min, 20 min after anesthesia induction, and end of surgery) and a between-subjects factor of groups (ondansetron vs. palonosetron). In contrast, for HR, Mauchly’s sphericity test showed a violation of the sphericity assumption (P = 0.04); thus, a one-way Wilk’s λ multivariate analysis of variance (MANOVA) was applied. The independent factor was each group (ondansetron vs. palonosetron), and the dependent variables were HR at each time point (Base, 5 min, 10 min, 15 min, 20 min after anesthesia induction, and end of surgery). To compare serial data at each time points, Student’s t-tests with Bonferroni correction (α = 0.05/7 = 0.0071) were used. Descriptive variables were presented as absolute number (%) and were analyzed using χ^2^ analyses or Fisher’s exact tests, as appropriate. A *P*-values < 0.05 was considered statistically significant. All statistical analyses were conducted using SPSS 26.0 (IBM Corp., Armonk, NY, USA).

## Results

**Fig 1:**
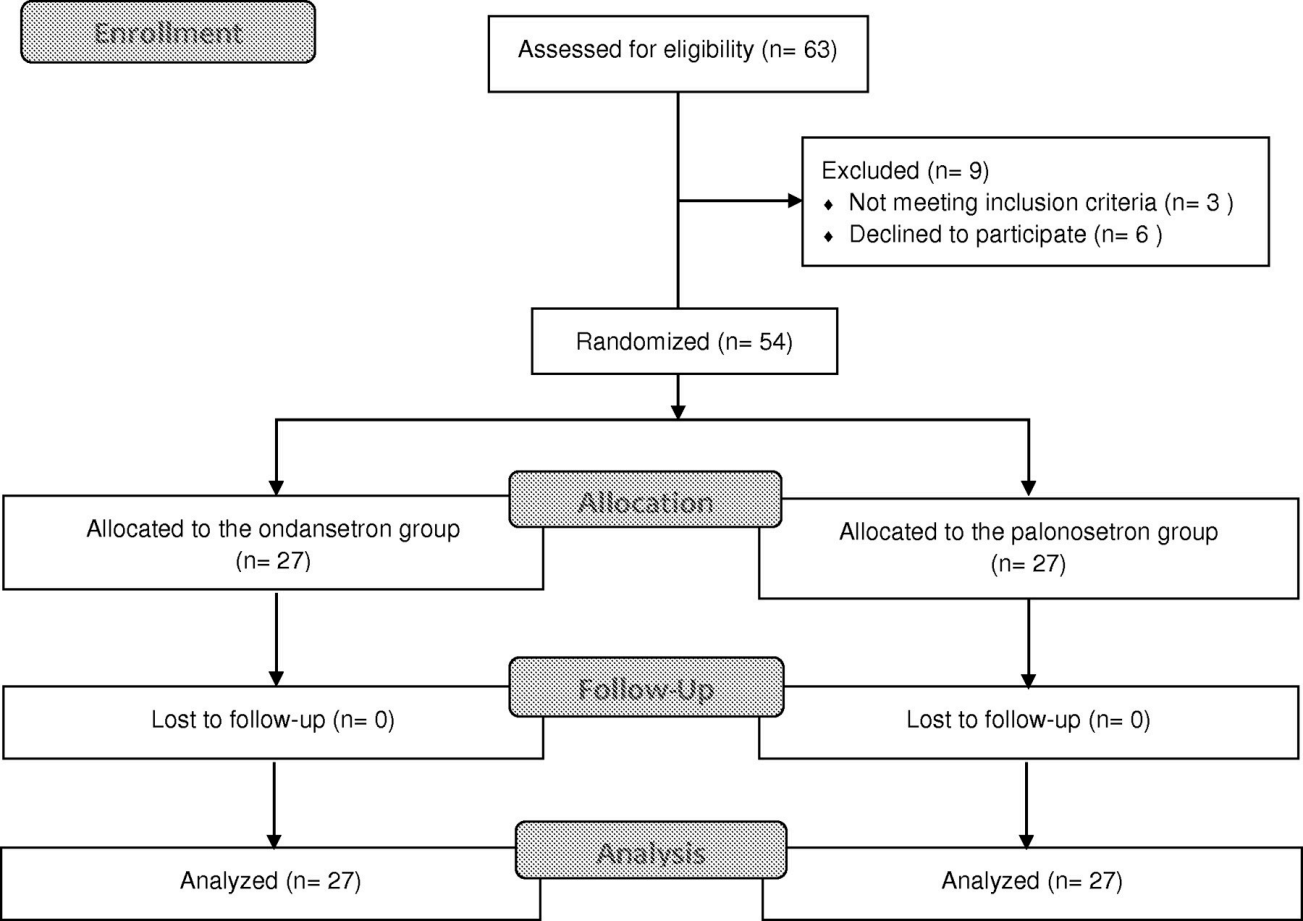
CONSORT flow diagram.

A total of 63 patients were assessed for eligibility. Of these, six patients opted not to participate, and three did not meet the inclusion criteria. A total of 54 patients subsequently allocated to groups, with no exclusions occurring thorough the study’s conclusion (Fig 1). Notably, there were no instances of spinal anesthesia failure or inadequate block that required an additional dose or sedation to achieve the desired sensory block level. Patient characteristics are shown in Table 1.

**Table 1:** Patient characteristics.

	Ondansetron (n = 27)	Palonosetron (n = 27)
Age (years)	35±3.8	34±4.5
Height (cm)	161.3±4.9	160.6±5.4
Weight (kg)	69.4±8.7	69.6±9.1
BMI (kg/m^2^)^a^	26.7±2.9	27.0±3.8
Gestational age (week)	38 (37–38) ±	38 (37–38)
Time from spinal to delivery (min)^b^	20 (18–24)	21 (19–24)
Time from incision to delivery (min)^c^	4 (2–6)	4 (2–7)

Data are expressed as the mean ± SD or median (interquartile range).

^a^ BMI refers to body mass index, which is the ratio of height to weight, expressed as kg/m^2^

^b^ “The time to delivery” refers to the duration from the administration of spinal anesthesia to the extraction of the fetus.

^c^ “The time from incision to delivery” refers to the period from skin incision to the extraction of the fetus.

**Fig 2:**
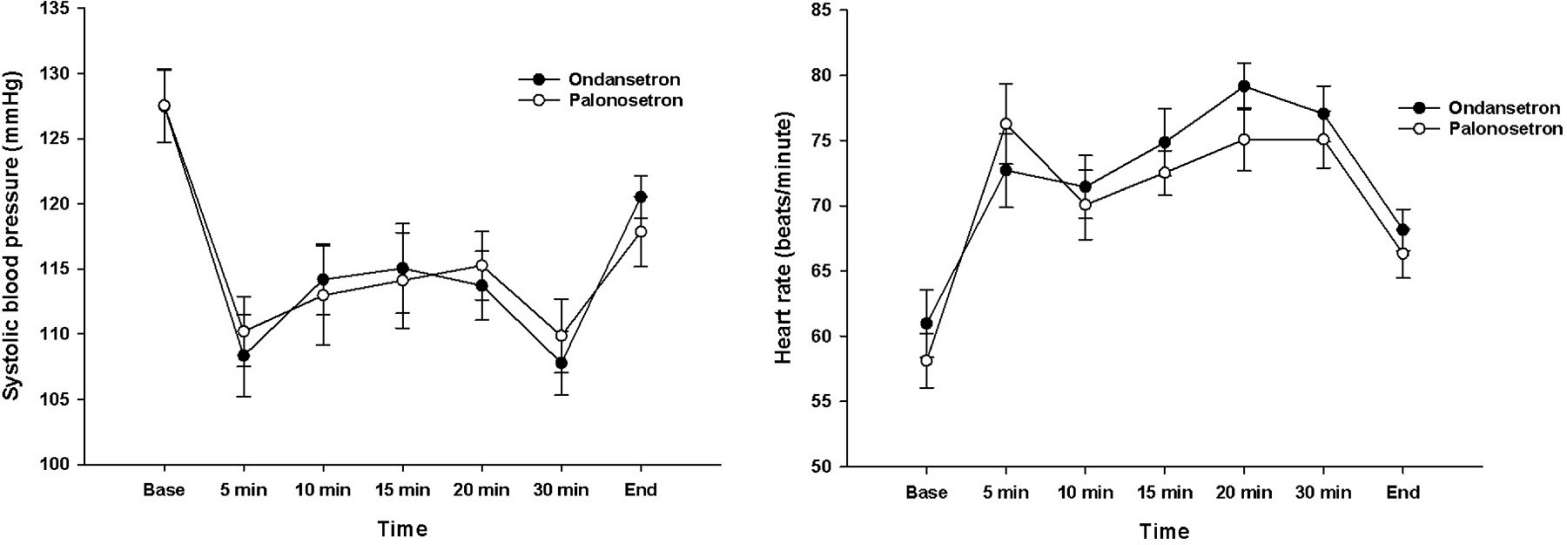
Hemodynamic changes between the ondansetron and palonosetron groups.

Table 2 presents a comprehensive overview of key outcomes. There were no differences between the two groups in total consumption of phenylephrine (*P* = 0.42). No significant hemodynamic alterations were noted between the two groups (Table 2). Analysis of SBP and HR changes throughout the surgical procedure also revealed no statistically significant differences between the groups (Fig 2). Results from RM-ANOVA showed no evidences of differences between groups (SBP; F[1, 52] = 0.001, *P* = 0.97, partial η2 < 0.001, HR; F[7, 46] = 0.732, *P* = 0.65: Wilk’s lamda = 0.900 partial η2 = 0.100).

**Table 2:** Primary, secondary, and other outcomes.

	Ondansetron (n = 27)	Palonosetron (n = 27)	Estimated Treatment Effect	*p*-value
**Requirements of phenylephrine**				
Continuous infusion dose (μg)	333.8±100.0	373.1±114.1	-39.3(-97.9 to 19.3)	0.18
Rescue dose (μg)	50 (0–100)	0 (0–100)	0.0(-0.0 to 50.0)	0.51
Total dose of phenylephrine (μg)^a^	387.5 (291.3–507.8)	428.0 (305.0–507.0)	-31.0(-107.0 to 45.0)	0.42
**Hemodynamic data** **Baseline**				
SBP (mmHg)	127.5±14.3	127.5±14.6	-0.1 (-8.0 to 7.8)	0.99
DBP (mmHg)	71.9±7.6	73.8±7.4	-1.9 (-6.0 to 2.2)	0.36
MBP (mmHg)	94.0±9.4	95.6±8.9	-1.7 (-6.7 to 3.3)	0.5
HR (bpm)	74.3 (67.3–85.7)	72.3 (69.0–85.7)	-0.7(-7.7 to 6.0)	0.84
**Lowest**				
SBP (mmHg)	96.1±17.0	98.5±15.9	-2.4 (-11.4 to 6.6)	0.6
DBP (mmHg)	50.5±9.2	50.6±9.5	-0.1 (-5.3 to 5.0)	0.95
MBP (mmHg)	68.0±11.1	70.3±11.7	-2.2 (-8.5 to 4.0)	0.47
HR (bpm)	61.0±13.5	58.1±10.7	2.9 (-3.8 to 9.5)	0.39
**Apgar score**				
1 min	9 (8–9)	9 (8–9)	-0.0(-1.0 to 0.0)	0.2
5 min	10 (9–10)	10 (9–10)	-0.0(-0.0 to 0.0)	0.24
**Postoperative nausea and vomiting**				
CR^b^, 2 hours after spinal anesthesia	24 (88.9%)	27 (100%)	0.9 (0.8 to 1.0)	0.24
CR, 24 hours after spinal anesthesia	22 (81.5%)	24 (88.9%)	0.9 (0.7 to 1.1)	0.7

Data are expressed as the mean ± SD or median (interquartile range).

Data representing the estimated treatment effect are expressed as follows: mean (95% confidence interval) for hemodynamic data and continuous infusion dose; median (95% confidence interval) for rescue dose and total dose of phenylephrine, as well as Apgar score; and relative risk (95% confidence interval) for CR.

^a^ Primary outcome

^b^ “Complete response (CR)” is defined as the absence of nausea and vomiting and no need for additional antiemetics. Data are presented in number (%).

Abbreviations: SBP, systolic blood pressure; DBP, diastolic blood pressure; MAP, mean arterial pressure; HR, heart rate; bpm, beats per minute; CR, complete response.

No difference in the incidence of bradycardia were observed (25.9% [7 of 27] in the ondansetron group compared to 27.8% [15 of 27] in the palonosetron group; *P =* 0.76). The rates of hypotension did not differ significantly between the groups (63% [17 of 27] in the ondansetron group versus 48.1% [13 of 27] in the palonosetron group; *P =* 0.27).

With respect to CR and the occurrence of nausea/vomiting, no significant differences were found between the two groups either within two hours following spinal anesthesia or 24 hours post-surgery (Table 2). Both groups were free from instances of shivering and adverse events.

## Discussion

In our study, we evaluated the efficacy of palonosetron versus ondansetron in reducing the use of phenylephrine for spinal anesthesia-induced hypotension during cesarean section. The results showed that there are no differences in the phenylephrine requirement.

After spinal anesthesia, the maternal systemic vascular resistance and blood pressure undergo rapid fluctuations within the initial 1–5 minutes [11]. Several mechanisms contribute to spinal anesthesia-induced hypotension. Notably, the most critical factor is the rapid onset of sympatholytic effects, which arise due to increased sensitivity of nerve fibers to local anesthetics during pregnancy [12]. Elevated sympathetic activity in pregnancy, compared to parasympathetic activity [12, 13], can lead to pronounced sympatholytic effects. This results in a shift toward parasympathetic predominance, manifesting as hypotension, bradycardia, nausea, and vomiting. The aortocaval compression by the gravid uterus further exacerbates the incidence and severity of hypotension [12, 14]. Since hypotension can endanger both the mother and fetus, maintaining optimal hemodynamic state is crucial.

The activation of the Bezold-Jarisch reflex involves both mechanoreceptors in the left ventricle and chemoreceptors. Mechanoreceptors are activated by the reduced venous return resulting from sympatholytic effects, while chemoreceptors are stimulated by serotonin acting on the 5-HT_3_ receptor located on vagal nerve endings in the left ventricle [15, 16]. This reflex contributes to bradycardia, reduced cardiac output, and exacerbation of spinal anesthesia-induced hypotension. Theoretically, 5-HT_3_ receptor antagonists could mitigate this reflex’s paradoxical activation, attenuate its induced hemodynamic changes, and thus potentially reduced the need for vasopressors. This theoretical framework has been supported by several prior studies [4–6, 8].

Palonosetron, the most recently developed second-generation 5-HT_3_ antagonist, has distinct properties compared to other 5-HT_3_ antagonists. Differing in chemical structure, it features a fused tricyclic ring system rather than the 3-substituted indole structure similar to serotonin seen in other 5-HT_3_ antagonists. Moreover, palonosetron has allosteric action with positive cooperativity and uniquely promotes receptor internalization inducing the long-term inhibition of the receptor, whereas other 5-HT_3_ receptor antagonists have competitive antagonistic effects with serotonin [17]. These unique attributes suggest potential advantages for palonosetron in preventing spinal anesthesia-induced hypotension, in addition to its role in mitigating nausea and vomiting. Previous study [8] have indicated that ramosetron, a more potent antagonist, effectively reduces spinal anesthesia-induced hypotension compared with ondansetron. Therefore, it was hypothesized that palonosetron might be more effective than ondansetron. Contrary to this expectation, our findings revealed no significant differences in the hemodynamic changes or phenylephrine requirements between the palonosetron and ondansetron groups. In addition to the total dose of phenylephrine used, no statistically significant difference was observed in the average dose calculated to account for the difference in the time until delivery between the palonosetron and ondansetron groups (0.27±0.05 μg/kg/min vs. 0.29±0.06 μg/kg/min, respectively).

The average dose in our ondansetron group was slightly higher than the effective dose in 50% of subjects (ED50) of a prophylactic phenylephrine infusion in the ondansetron group (0.24 μg/kg/min) for preventing hypotension in Xiao’s study [7]. However, it was lower than that of the Xiao’s control group (0.32 μg/kg/min). The ED50 value of the ondansetron group in Xiao’s study was lower than the average dose in the palonosetron group in our study. Several factors could account for this discrepancy, such as variations in the study population, differences in study design, and anesthetic technique employed, including the specific types and doses of local anesthetic or opioids used during spinal anesthesia. Another contributing factor could be that the vasodilatory effects induced by subarachnoid blockade had a more pronounced impact on blood pressure than the attenuation of the Bezold-Jarisch reflex by the 5-HT_3_ receptor antagonists. Given the ongoing debate over the impact of 5-HT_3_ receptor antagonists on hemodynamic changes after spinal anesthesia [18–21], prophylaxis for spinal anesthesia-induced hypotension should not be the sole purpose for drug selection among 5-HT_3_ receptor antagonists.

The etiology of nausea and vomiting following cesarean section under spinal anesthesia is multifactorial, commonly attributed to factors such as hypotension, uterine exteriorization, peritoneal traction, perioperative opioids, and vagal activity [14, 22, 23]. 5-HT_3_ receptor antagonists are the first-line treatment for preventing these symptoms [9]. Palonosetron is considered more effective in awake patients under spinal anesthesia due to its higher receptor binding affinity and longer plasma half-life [24–26]. When considering the theoretical basis that blocking of receptors in the chemoreceptor trigger zone occurs before the arrival of stimuli, administering palonosetron before induction of anesthesia may be more advantageous for preventing PONV and ensuring hemodynamic stability following spinal anesthesia, compared with other 5-HT3 antagonists typically administered before the end of surgery [9]. Contrary to these expectations, our results showed no significant difference in the CR rate for PONV between the groups. It is important to note that our study’s sample size was calculated based on the dose of phenylephrine used, and all participants received a regimen of intravenous patient-controlled analgesic that included a mixture of fentanyl and ondansetron. These variables could have influenced the study outcomes, warranting cautious interpretation.

Our study has several limitations. First, we did not include a control group. We initially formulated our research based on the assumption that ondansetron attenuates spinal anesthesia-induced hypotension in cesarean section, as suggested by previous study [7]. Therefore, we could not directly assess the true effectiveness of 5-HT_3_ antagonists in alleviating hypotension when compared to a control group. Second, there is a difference between the standard deviation used in sample size calculations and the standard deviation of the actual study results. These differences may have influenced our results, which found that the difference in outcomes was larger than the MCID but not statistically significant. Therefore, careful consideration is warranted in interpreting the clinical significance, and further research with increased statistical power may be necessary. Third, our study was neither designed nor adequately powered to explore clinical outcomes of nausea and vomiting, either intraoperatively or postoperatively. The impact of the treatment on these symptoms might have been underestimated. Future studies with larger sample sizes are necessary to validate these effects.

## Conclusion

In patients undergoing cesarean section with spinal anesthesia, palonosetron did not demonstrate superior efficacy in mitigating or preventing spinal anesthesia-induced hypotension when compared to ondansetron.

## Supporting information

S1 Table The CONSORT 2010 checklist. (DOC)

S1 File Dataset. (XLSX)

S2 File Study protocol (Original language). (PDF)

S3 File Study protocol (English version). (PDF)
