# Supplementary material for: Effect of intravenous palonosetron on hypotension induced by spinal anesthesia for cesarean section: A randomized controlled trial
Source: PLoS One. 2024 Jun 25;19(6):e0305913. doi: 10.1371/journal.pone.0305913 (PMC11198823; doi:10.1371/journal.pone.0305913)
Supplement: S2 File — (PDF) [file pone.0305913.s003.pdf]

Palonosetron 정맥주사가 제왕절개를 위한 척추마취 시행에 따른 저혈압 발생의 예방 목적으로 사용되는 phenylephrine 사용량에 미치는 영향: 무작위 배정 임상시험

Effect of intravenous palonosetron on hypotension induced by spinal anesthesia for cesarean section: a randomized controlled trial

Version No 1.3

책임연구자 소속: 중앙대학교병원 마취통증의학과 책임연구자 이름: 백 종화

연구 개요

연구제목	(국문) Palonosetron 정맥주사가 제왕절개를 위한 척추마취 시행에 따른 저혈압 발생에 미치는 영향: 무작위 배정 임상시험
	(영문) Effect of intravenous palonosetron on hypotension induced by spinal anesthesia for cesarean section: a randomized controlled trial
책임연구자	마취통증의학과 교수 백종화
연구비 지원기관	없음

연구 목적	제왕절개를 시행받는 환자에서 Palonosetron 정맥투여가 척추마취에 의한 저혈압 발생에 미치는 영향을 phenylephrine 사용량 비교를 통해 알아보고자 한다.
연구 설계	전향적 무작위 배정 연구
연구 기간	IRB 승인일 ~ 1 년
연구 대상<br>(시험약 등)	ASA 1~2 제왕절개 수술이 예정된 단태아 산모<br>Palonosetron 0.075mg IV 투여
연구 대상자 수	두 군에서 각 군당 27 명으로 총 54 명
취약한 연구대상자	해당사항 없음
연구 방법	척추마취하 제왕절개 수술 예정인 환자들 중에 본 연구에 동의한 환자들을 대상으로 무작위배정표에 따라 대조군 (control group, group C)과 시험군 (palonosetron group, group P)으로 배정한다.<br>군 배정을 담당하는 연구자는 해당 군에 따라 약물을 준비하여 대상자의 번호로만 기입된 봉투에 동봉한 후 마취 시행을 담당하는 연구자에게 전달한다 (group C: ondansetron 4mg/2ml, group P: palonosetron 0.075mg/1.5ml 와 Saline 0.5ml 혼합하여 total 2ml). 군배정과 약물 준비에 관여한 연구자는 다른 연구 단계에는 개입되지 않도록 배제한다.<br>척추마취는 연구의 다른 단계에 개입되지 않는 마취통증의학과 의사가 담당하도록 한다. 척추 마취 시행 10 분 전 각 군에 해당되는 약물을

	정맥주사하고 비침습적 혈압계를 이용하여 3 분간격으로 3 번의 혈압을 측정하여, 그 평균값을 baseline value 로 기재한다. 척추마취 시행 후 태아 만출 시까지 1 분 간격으로 혈압을 측정한다. 척추 마취 시행후 phenylephrine infusion 을 0.24mcg/kg/min 으로 주입하기 시작하며, baseline value 를 기준으로 <80% 시 phenylephrine 50mcg 을 bolus 주입을 하고, >120% 시 infusion 을 중지한다.<br>Bradycardia <55 beats/min 인 경우에는 0.5mg atropine 을 투여한다.<br>태아만출 시까지 사용된 phenylephrine 의 총량을 기재하도록 하며, neonatal outcome 으로 1 분, 5 분 Apgar score 를 기록한다.
유효성 평가	두 군간의 phenylephrine 사용량을 비교한다.
안전성 평가	Palonosetron 을 PONV 예방 목적으로도 마취 시행시 이미 안전하게 사용되고 있는 약으로 해당 약물 주입에 따른 위해는 없을 것으로 판단된다.
기대효과 및 예상결과	Palonosetron 과 같은 5HT-3 receptor antagonist 인 ondanseron 과 ramosetron 이 척추마취에 의한 저혈압 발생의 위험을 줄인다는 선행연구가 있는 만큼, palonosetron 또한 이에 효과를 보이리라 기대된다. 선행 연구에서 사용된 ondansetron 과 ramosetron 이 PONV 예방 목적으로는 수술 종료 30 분 전에 투여하는 것이 좋다는 것과 달리 palonosetron 은 마취유도시에 투여하는 것이 좋다는 것을 고려했을 때, palonosetron 이 PONV 예방에도 더욱 좋은 효과를 보일 것으로 사료된다.

연구계획서

1. **연구 제목** Palonosetron 정맥주사가 제왕절개를 위한 척추마취 시행에 따른 저혈압 발생에 미치는 영향: 무작위 배정 임상시험

2. **연구의 실시기관 명칭 및 주소** 중앙대학교병원: 서울특별시 동작구 흑석로 102

3. **연구 의뢰기관** 해당 없음

4. **연구비 지원기관 명칭 및 주소** 해당 없음

5. **예상연구기간**

   IRB 승인 일 이후 1 년

6. **연구 대상 질환**

   척추마취 하 제왕절개 수술을 필요로 하는 질환

7. **연구의 배경 및 목적**

   1) **연구 배경**

제왕절개 수술에 있어 척추마취는 주로 사용되는 마취 방법이나, 이는 혈역학적 변화를 초래할 수 있다. 주로 발생되는 혈역학적 변화로는 척추마취에 따른 교감신경 차단으로 인한 전신 혈관저항 감소와 심박출량 감소에 따른 저혈압과 상대적 부교감신경 항진, Bezold-Jarisch reflex, baroreceptor activity 증가에 따른 서맥이 있다. 따라서 이에 인해 척추마취 후 초래되는 저혈압 등의 혈역학적 변화를 막기 위해 통상적으로 phenylephrine 이 사용된다.

Phenylephrine 은 direct-acting sympathomimetic amine 으로 post-synaptic alpha-1 adrenergic receptor agonist 로 작용하며 adrenaline 과 ephedrine 의 강력한 혈관 수축 작용과 연관이 있는 약물이다.

5-HT3 receptor antagonist 는 serotonin receptor sensitive chemoreceptor 에 의해 trigger 되는 Bezold-Jarisch reflex 에 영향을 주어 5-HT3 induced bradycardia 를 감소시키고 혈압 변동을 개선시킨다고 보고된 바 있다.

선행 연구에 사용된 ondansetron 은 serotonin 5-HT_3 receptor antagonist 로, 위장관의 vagal affrents 와 chemoreceptor trigger zone 에 있는 세로토닌 수용체에 결합하여 구역과 구토를 완화시켜주는 작용을 한다.

Palonosetron 은 carbazole 유도체로, selective serotonin receptor antagonist 이다. Palonosetron 은 5-HT3 receptors 에 작용하여 serotonin 의 경쟁적 길항제로 작용하게 된다. Palomosetron 은 선행 연구에서 사용된 ondansetron 과 ramosetron 과 같은 기존의 5-HT3 antagonist 들 보다 potent 하고 작용 기간도 길기 때문에 이에 따른 개선효과도 클 것이라 사료되지만, 아직 palonosetron 의 주사에 따른 영향에 대한 연구는 보고된 바 없다. 따라서 본 연구는 palonosetron 의 이러한 효과를 phenylephrine 의 사용량 비교를 통해 보다 정량적으로 선행 연구에 사용된 ondansetron 과 비교하고자 한다.

2) 연구 가설 및 목적

Palonosetron 투여를 통해 척추마취에 의한 저혈압 발생 위험을 감소시킬 것으로 생각되며, 이는 태아 안녕에도 도움이 될 것으로 기대할 수 있다. 따라서 본 연구를 통해 이를 입증하고 한다.

8. 임상연구용 의약품 및 의료기기 코드명(또는 주성분의 일반명), 원료약품의 분량, 제형 등(대조약 포함)

WIZPAL1 알록시주 (Palonosetron) 0.075mg/1.5ml

WIZOD4 온단트주(Ondansetron) 4mg/2ml

9. 연구대상자의 선정 기준, 제외기준, 목표한 대상자 수 및 산출 근거

1) 선정기준

- ASA 2 의 단태아 산모 중 정규로 척추마취 하
  제왕절개 수술를 받는 환자

2) 제외기준

- 18 세 이하, 65 세 이상
- 체중 45kg 이하, 100kg 이상
- ASA 3 이상
- 뇌질환 또는 정신과적 질환이 있거나 관련 약물을 복용하는 환자
- 심각한 심혈관계, 신장, 간 혈액학적 이상이 있는 환자
- 응급 제왕절개 수술
- 임신성 고혈압, Pre-eclampsia, Eclampsia, 전치태반 등의 임신 관련 합병증을 동반한 산모
- 다태아 산모

- 태아에게 기형이 있거나 1500g 미만의 극소저체중출생아(Very Low Birth Weight Infant)가 예상되는 경우
- 산모가 척추마취를 거부하는 경우

3) 목표한 대상자 수

54 명

4) 대상자 수 산출근거

▶ 산출 프로그램

PASS software, version 11 (NCSS, Kaysville, UT, USA)

▶ 산출 조건 및 공식

본 연구의 primary endpoint 는 phenylephrine 의 사용량이다. 이 전 연구에서 Ondansetron 을 사용한 군에서 phenyephrnie 의 사용량은 316.5 ± 25.9 이♘다. Ondansetron 과 ramosetron 의 spinal 전 후 SBP 의 변화량은 각각 25.1%, 17.9%이♘다. 따라서 palonosetron 의 군에서 phenlyephrine 의 사용량의 표준편차가 ondansetron 과 같고, 사용량이 7% 감소한다는 가정하에, α 오차를 5%, β 오차를 20%로 받아들여 계산한 결과 각 군의 시험자 수는 24 명이♘다.

Two-Sample T-Test Power Analysis

Numeric Results for Mann-Whitney Test (Normal Distribution)

Null Hypothesis: Mean1=Mean2. Alternative Hypothesis: Mean1≠Mean2

The standard deviations were assumed to be known and unequal.

Power	N1	N2	Allocation Ratio	Alpha	Beta	Mean1	Mean2	S1	S2
0.80978	24	24	1.000	0.05000	0.19022	316.5	294.3	25.9	25.9

References

Machin, D., Campbell, M., Fayers, P., and Pinol, A. 1997. Sample Size Tables for Clinical Studies, 2nd Edition. Blackwell Science. Malden, MA.

Zar, Jerrold H. 1984. Biostatistical Analysis (Second Edition). Prentice-Hall. Englewood Cliffs, New Jersey.

Al-Sunduqchi, Mahdi S. 1990. Determining the Appropriate Sample Size for Inferences Based on the Wilcoxon Statistics. Ph.D. dissertation under the direction of William C. Guenther, Dept. of Statistics, University of Wyoming, Laramie, Wyoming.

Report Definitions

Power is the probability of rejecting a false null hypothesis. Power should be close to one.
N1 and N2 are the number of items sampled from each population. To conserve resources, they should be small.
Alpha is the probability of rejecting a true null hypothesis. It should be small.
Beta is the probability of accepting a false null hypothesis. It should be small.
Mean1 is the mean of populations 1 and 2 under the null hypothesis of equality.
Mean2 is the mean of population 2 under the alternative hypothesis. The mean of population 1 is unchanged.
S1 and S2 are the population standard deviations. They represent the variability in the populations.

Summary Statements

Group sample sizes of 24 and 24 achieve 81% power to detect a difference of 22.2 between the null hypothesis that both group means are 316.5 and the alternative hypothesis that the mean of group 2 is 294.3 with known group standard deviations of 25.9 and 25.9 and with a significance level (alpha) of 0.05000 using a two-sided Mann-Whitney test assuming that the actual distribution is normal.

여기에 탈락율(drop-out rate) 10%를 적용하여 군당 27 명의 환자를 대상으로 연구를 진행하기로 하였다.

5) 연구 대상자 모집 계획

중앙대학교병원에서 정규 수술로 척추마취하 제왕절개 수술이 계획된 환자 중 본 연구에 동의한 자를 연구 대상자로 선정한다. 별도의 모집공고문은 필요로 하지 않는다.

10. 연구 방법

1) 구체적인 연구방법

척추마취하 제왕절개 수술 예정인 환자들 중에 본 연구에 동의한 환자들을 대상으로 무작위배정표에 따라 대조군 (control group, group C)과 시험군 (palonosetron group, group P)으로 배정한다.

군 배정을 담당하는 연구자는 해당 군에 따라 약물을 준비하여 대상자의 번호로만 기입된 봉투에 동봉한 후 마취 시행을 담당하는 연구자에게 전달한다 (group C: ondansetron 4mg/2ml, group P: palonosetron 0.075mg/1.5ml 와 Saline 0.5ml 혼합하여 total 2ml). 군배정과 약물 준비에 관여한 연구자는 다른 연구 단계에는 개입되지 않도록 배제한다.

척추마취는 연구의 다른 단계에 개입되지 않는 마취통증의학과 의사가 담당하도록 한다. 척추 마취 시행 10 분 전 각 군에 해당되는 약물을 정맥주사하고 비침습적 혈압계를 이용하여 3 분간격으로 3 번의 혈압을 측정하여, 그 평균값을 baseline value 로 기재한다. 척추마취 시행 후 태아 만출 시까지 1 분 간격으로 혈압을 측정한다. 척추 마취 시행후 phenylephrine infusion 을 0.24mcg/kg/min 으로 주입하기 시작하며, baseline value 를 기준으로 <80% 시 phenylephrine 50mcg 을 bolus 주입을 하고, >120% 시 infusion 을 중지한다.

Bradycardia <55 beats/min 인 경우에는 0.5mg atropine 을 투여한다.

태아만출 시까지 사용된 phenylephrine 의 총량을 기재하도록 하며, neonatal outcome 으로 1 분, 5 분 Apgar score 를 기록한다

2) 비교군 설정 및 무작위 배정 방법

미리 제작된 Randomization table 의 순서에 따라 1:1 로 무작위 배정을 실시

3) 시험약 투여·사용량, 투여·사용 방법, 병용 요법, 대조약 사용시 그 선택사유

Palonosetrron 군: palonosetron 0.075mg(1.5ml) 와 Saline 0.5ml IV 투여

Control 군: ondansetron 4mg/2ml IV 투여

4) 관찰항목, 임상검사항목 및 관찰검사방법

- 1 차 관찰 항목
  - 태아만출 시점까지 사용된 phenylephrine 의 총량
- 2 차 관찰 항목
  - 관찰기간 동안에 가장 낮은 SBP, DBP, MBP, HR
  - Hypotesion 발생 유무
  - Bradycardia 발생 유무
  - Nausea/vomiting 발생 유무
  - Sheivering 발생 유무
- 기타 관찰 항목
  - Age, Sex, Weight, Height, BMI, ASA 등급, 병력, gestational age
  - 척추마취 시행 후 30 분째에 해당되는 sensory block level
- 관찰 검사 방법
  - Vital sign 을 비침습적 모니터링을 통해 측정한 값을 이용한다.
  - Phenylephrine 총 사용량은 태아만출 시점에 infusion pump 에 기록되는 총 사용량을 기재한다.

5) 유효성 평가기준, 평가방법

두 군간의 phenylephrine 총 사용량을 비교한다.

6) 기존 치료 및 연구와의 차별점

기존의 연구에서 항구토제로 사용되는 ondansetron 과 ramosetron 이 척추마취에 의한 저혈압 발생을 줄인다는 보고가 있다. 하지만 차세대 약물인 palonosetron 에 대한 연구는 아직 보고된 바가 없다. 또한 PONV 예방 목적의 사용에서 ondansetron 과 ramosetron 은 수술 종료 시점으로부터 30 분 전에 투여하는 것이 좋다는 것으로 보았을 때 PONV 감소 효과는 경감될 수 있다는 문제가 있다. 차세대 항구토제인 palonosetron 은 PONV 예방 목적의 투여시점이 마취 유도 직전이라는 점에서 볼 때 해당 약물이 척추마취에 의한 저혈압 발생을 줄인다면, 두 가지 효과를 기대할 수 있기에 더욱 이상적이라 할 수 있다.

7) 연구대상자의 이익과 위험

Palonosetron 의 사용을 통해 PONV 예방 효과를 볼 수 있을 뿐만 아니라, palonosetron 사용에 따른 척추마취 후 저혈압의 발생이 감소된다면, 보다 안전한 마취관리가 이루어질 수 있으며, 이는 태아 안녕에도 도움이 될 것이다.

일반적으로 안전하게 사용되고 있는 용량의 palonosetron 을 사용하기 때문에 다른 위험요인은 없다.

8) 중지·탈락 기준

- 중도에 연구 참여를 거부한 자
- 척추마취가 불완전하여 마취계획이 변경되는 등 예상하지 못한 상황 발생하는 경우

9) 부작용을 포함한 안전성의 평가기준, 평가 방법 및 보고 방법

- Palonosetron 의 사용과 관련하여 보고된 부작용
- 발생한 부작용에 대해서 치료를 시행하고 환자의 건강에 위해가 되는 경우 책임연구자는 중앙대학교병원 IRB 에 보고한다.

10) 자료안전성 모니터링 계획(DSMP)

연구 책임자를 자료안전담당자로 하여 월 1 회 연구 자료를 모아 검토하고 관리하여 지속적인 안전성에 대한 모니터링을 시행하여 자료의 완전성을 보증하고 연구 대상자의 안정성을 확보할 것이다.

11) 자료 분석 및 통계 분석 방법

연속형 자료에서 Shapiro-Wilk test 로 수집된 자료들의 정규 분포성을 검증하고 정규분포를 하는 경우 t-test 를, 정규분포를 하지 않는 경우 Mann-whiteny U test 를 시행한다. 비연속적 자료의 경우 chi-squared analysis 나 Fischer's exact test 를 시행한다. p 값이 0.05 이하일 경우 통계적으로 유의하다고 한다. 통계적 분석은 SPSS 15.0 을 이용하여 분석하며 정규분포를 하는 자료들은 평균 ± 표준편차로 표시하고, 정규분포를 하지 않은 자료는 중간값(사분위범위)로 표시한다.

12) 연구수행일정표

IRB 승인일 ~ 9 개월: data 수집

9 개월 – 1 년: 데이터 분석 및 논문 작성

11. 연구대상자의 안전보호를 위한 대책

1) 연구의 윤리성 확보를 위한 기본 방안

본 임상 연구는 중앙대학교병원의 생명윤리심의위원회의 심의를 거쳐 실시되며 IRB 승인 후 연구가 진행될 예정이다. 본 연구에 참여하는 대상자의 정보는 오직 임상 연구에 참여하는 의료진에 의해 연구의 목적으로만 사용되도록 한다. 향후 임상시험의 결과가 출판되는 경우에도 해 당 연구 대상자의 신원은 절대 노출되지 않도록 할 것이다.

2) 연구대상자의 동의 과정

연구책임자 혹은 공동연구자를 통하여 직접 동의를 받게 될 것이고 고위험군을 제외한 환자 본인에게 동의를 받게 될 것이다. 연구 설명 및 동의 취득은 수술 전날 강제성 없이 이루어질 것이고 연구 비참여로 인한 부당한 영향은 없을 것을 미리 공지할 예정이다. 설명은 환자 및 보호자가 이해하기 쉽게 이루어질 것이고 동의서 서명은 직접 환자 혹은 보호자에게 받게 될 것이다

3) 연구대상자의 보상 방안

임상시험의 참여로 인한 대상자의 신체적 손상이 발생할 경우, 금전적 보상에 대하여 확정되기 전이라도 임상시험책임자(담당자)를 통한 적절한 치료 또는 치료 기회를 우선적으로 제공한다.

4) 연구대상자의 개인정보보호 방안

연구대상자의 개인 정보는 연구책임자 및 공동연구자만 열람할 수 있도록 암호화된 파일로 저장될 것이며 연구가 종료된 시점으로부터 약 3 년간 보관 후 파기할 것이다.

5) 취약한 연구대상자를 포함하는 경우 추가적인 보호조치 방안

해당사항 없음

12. 인체유래물의 보관 및 폐기 방법

해당사항 없음.

13. 참고 문헌

1) Chattopadhyay S, Goswami S. Palonosetron Versus Ramosetron Prophylaxis for Control of Postoperative Nausea and Vomiting after Cesarean Delivery under Spinal Anesthesia. Journal of obstetrics and gynaecology of India. 2015;65(1):28-33.

2) Shin HJ, Choi ES, Lee GW, Do SH. Effects of Preoperative Serotonin-Receptor-Antagonist Administration in Spinal Anesthesia-Induced Hypotension: A Randomized, Double-blind Comparison Study of Ramosetron and Ondansetron. Regional anesthesia and pain medicine. 2015;40(5):583-8.

3) Varshney RK, Garg M, Kapoor K, Jheetay GS. The role of ramosetron in the prevention of post-spinal shivering in obstetric patients. A prospective randomized double blind study. Romanian journal of anaesthesia and intensive care. 2019;26(1):37-43.

4) Xiao F, Wei C, Chang X, Zhang Y, Xue L, Shen H, et al. A Prospective, Randomized, Double-Blinded Study of the Effect of Intravenous Ondansetron on the Effective Dose in 50% of Subjects of Prophylactic Phenylephrine Infusions for Preventing Spinal Anesthesia-Induced Hypotension During Cesarean Delivery. Anesthesia and analgesia. 2020;131(2):564-9.
