# Supplementary material for: Effect of intravenous palonosetron on hypotension induced by spinal anesthesia for cesarean section: A randomized controlled trial
Source: PLoS One. 2024 Jun 25;19(6):e0305913. doi: 10.1371/journal.pone.0305913 (PMC11198823; doi:10.1371/journal.pone.0305913)
Supplement: S3 File — (PDF) [file pone.0305913.s004.pdf]

Palonosetron 정맥주사가 제왕절개를 위한 척추마취 시행에 따른 저혈압 발생의 예방 목적으로 사용되는 phenylephrine 사용량에 미치는 영향: 무작위 배정 임상시험

Effect of intravenous palonosetron on hypotension induced by spinal anesthesia for cesarean section: a randomized controlledtrial

Version No 1.3

Researcher Affiliation: Department of Anesthesiology and Pain Medicine, Chung-Ang University Hospital
Principal Investigator: Chong Wha Baek

Research Overview

Study title	(Korean) Palonosetron 정맥주사가 제왕절개를 위한 척추마취 시행에 따른 저혈압 발생에 미치는 영향: 무작위 배정 임상시험
	(English) Effect of intravenous palonosetron on hypotension induced byspinal anesthesia for cesarean section: a randomized controlled trial
Principal Investigator	Chong Wha Baek, M.D., Ph.D.
Funding	None

Study purpose	The purpose of this study was to investigate the effect of intravenous administration of Palonosetron on hypotension caused by spinal anesthesia in patients undergoing caesarean section by comparing the amount of phenylephrine used.
Study design	Prospective randomized controlled trial
Study period	1 year from the date of IRB approval
Subject of study (including drug)	Singleton mothers with ASA 1 or 2 scheduled for caesarean section<br>(Palonosetron 0.075mg IV or Ondansetron 4mg IV)
Number of study subjects	there are 27 patients per group (total 54 patients)
Vulnerable research subjects	None

Study methods	Among patients scheduled for Cesarean section under spinal anesthesia, those who agreed to participate in this study will be randomly assigned to a control group (group C) and a palonosetron group (group P) according to a random allocation table.<br><br>The researcher in charge of group allocation will prepare the medication according to the assigned group and place it in an envelope with only the subject's number written on it. The envelope will be handed over to the researcher in charge of anesthesia, who will not be involved in any other stages of the study (group C: ondansetron 4mg/2ml, group P: palonosetron 0.075mg/1.5ml mixed with saline 0.5ml to a total of 2ml).<br><br>Spinal anesthesia will be performed by an anesthesiologist specializing in anesthesia and pain medicine who will not be involved in other stages of the study. The medication corresponding to each group will be administered intravenously 10 minutes before spinal anesthesia. Blood pressure will be measured at 3-minute intervals using a non-invasive blood pressure monitor, and the mean value will be recorded as the baseline value.<br><br>After spinal anesthesia, blood pressure will be measured at 1-minute intervals until delivery of the fetus. Phenylephrine infusion will be started at a rate of 0.24mcg/kg/min, and if the blood pressure falls below 80% of the baseline value, a phenylephrine bolus of 50mcg will be administered. If the blood pressure exceeds 120% of the baseline value, the infusion will be stopped.<br><br>If the heart rate falls below 55 beats/min, 0.5mg of atropine will be administered. The total amount of phenylephrine used until delivery of the fetus will be recorded, and the 1-minute and 5-minute Apgar scores will be recorded as neonatal outcomes.

Efficacy evaluation	Compare the total amount of phenylephrine used between the two groups.
Safety assessment	Palonosetron is already safely used for anesthesia even for the purpose of preventing PONV, and it is judged that there is no harm from injecting the drug.
Expected effects and expected results	As there are previous studies showing that ondanseron and ramosetron reduce the risk of hypotension caused by spinal anesthesia, palonosetron is also expected to have similar effect. As for the timing of administration, while the ondansetron and ramosetron were recommended to be given 30 minutes prior to the end of surgery for the purpose of preventing PONV (postoperative nausea and vomiting), it is believed that palonosetron will show even better effects in PONV prevention if administered during anesthesia induction.

Study protocol

1. **Study title** Effect of intravenous palonosetron on hypotension induced byspinal anesthesia for cesarean section: a randomized controlled trial

2. **Name and address of research institute**

Chung-Ang University Hospital, 84 Heukseok-ro, Dongjak-gu, Seoul 06911, Republic of Korea

3. **Institution requesting research** None

4. **Name and address of research fund support institution** None

5. **Study period**
   1 year from the date of institutional review board (IRB) approval

6. **Study target disease**
   Diseases requiring cesarean section under spinal anesthesia

7. **Background and purpose of the study**
   1) **Background**

Spinal anesthesia is the most commonly used anesthesia method for caesarean section, but it can cause hemodynamic changes. The main hemodynamic changes include decreased systemic vascular resistance due to sympathetic blockade following spinal anesthesia, hypotension and relative parasympathetic hyperactivity due to reduced cardiac output, and bradycardia due to increased Bezold-Jarisch reflex and baroreceptor activity. Therefore, phenylephrine is usually used to prevent hemodynamic changes such as hypotension caused after spinal anesthesia.
Phenylephrine is a direct-acting sympathomimetic amine that acts as a post-synaptic alpha-1 adrenergic receptor agonist and is related to the potent vasoconstrictor action of adrenaline and ephedrine.
It has been reported that 5-HT3 receptor antagonists reduce 5-HT3-induced bradycardia and improve blood pressure fluctuations by influencing the Bezold-Jarisch reflex triggered by serotonin receptor sensitive chemoreceptors.
Ondansetron, used in previous studies, is a serotonin 5-HT3 receptor antagonist that binds to serotonin receptors in the vagal affrents and chemoreceptor trigger zones of the gastrointestinal tract to relieve nausea and vomiting.
Palonosetron is a carbazole derivative and is a selective serotonin receptor antagonist. Palonosetron acts as a competitive antagonist of serotonin by acting on 5-HT3 receptors. Since Paloosetron is more potent than other 5-HT3 antagonists such as ondansetron and ramosetron used in previous studies and has a longer duration of action, it is believed that it will be more effective. However, studies on the effects of palonosetron have not yet been reported. Therefore, this study aims to quantitatively compare these effects of palonosetron with ondansetron used in previous studies by comparing the amount of phenylephrine used.

2) Hypothesis and purpose

Palonosetron administration is thought to reduce the risk of developing hypotension due to spinal anesthesia, which can be expected to benefit fetal well-being. Therefore, this study proves it.

8. Code names of drugs and medical devices for study (or generic names of active ingredients), quantity of raw materials, dosage form, etc. (including comparators)

WIZPAL1 Aloxi injection (Palonosetron) 0.075mg/1.5ml
WIZOD4 Ondant injection (Ondansetron) 4mg/2ml

9. Sugject eligibility, target number of subjects and basis for calculation

1) Inclusion criteria

- Single pregnancy mother sundergoing elective cesarean section under spinal anesthesia

2) Exclusion criteria

- Under 18 years old, over 65 years old
- Weight 45kg or less, 100kg or more
- ASA 3 or more
- Patients with brain or psychiatric disorders or taking related drugs
- Patients with severe cardiovascular, kidney, liver, or hematologic abnormalities
- Emergency cesarean section
- Gestational hypertension
- Pre-eclampsia, eclampsia, mothers with pregnancy-related complications such as placenta previa, multiple-pregnancy, malformation in the fetus or when a very low birth weight infant weighing less than 1500 g is expected
- When a patient refuses spinal anesthesia

3) Target Number

54

4) Basis for calculating the sample size

▶ Calculation program

PASS software, version 11 (NCSS, Kaysville, UT, USA)

▶ Calculation conditions and formulas:

The primary endpoint of this study is the usage of phenylephrine. In the previous study, the usage of phenylephrine in the group that used Ondansetron was 316.5 ± 25.9. The changes in SBP before and after spinal injection of Ondansetron and Ramosetron were 25.1% and 17.9%, respectively. Therefore, assuming that the standard deviation of phenylephrine usage in the palonosetron group is the same as that in the ondansetron group and that the usage decreases by 7%, the number of test subjects in each group was calculated assuming a type I error of 5% and a type II error of 20%, resulting in 24 subjects per group. After applying a drop-out rate of 10%, the study was conducted on 27 patients per group.

Two-Sample T-Test Power Analysis

Numeric Results for Mann-Whitney Test (Normal Distribution)

Null Hypothesis: Mean1=Mean2. Alternative Hypothesis: Mean1≠Mean2

The standard deviations were assumed to be known and unequal.

Power	N1	N2	Allocation Ratio	Alpha	Beta	Mean1	Mean2	S1	S2
0.80978	24	24	1.000	0.05000	0.19022	316.5	294.3	25.9	25.9

References

Machin, D., Campbell, M., Fayers, P., and Pinol, A. 1997. Sample Size Tables for Clinical Studies, 2nd Edition. Blackwell Science. Malden, MA.

Zar, Jerrold H. 1984. Biostatistical Analysis (Second Edition). Prentice-Hall. Englewood Cliffs, New Jersey.

Al-Sunduqchi, Mahdi S. 1990. Determining the Appropriate Sample Size for Inferences Based on the Wilcoxon Statistics. Ph.D. dissertation under the direction of William C. Guenther, Dept. of Statistics, University of Wyoming, Laramie, Wyoming.

Report Definitions

Power is the probability of rejecting a false null hypothesis. Power should be close to one.
N1 and N2 are the number of items sampled from each population. To conserve resources, they should be small.
Alpha is the probability of rejecting a true null hypothesis. It should be small.
Beta is the probability of accepting a false null hypothesis. It should be small.
Mean1 is the mean of populations 1 and 2 under the null hypothesis of equality.
Mean2 is the mean of population 2 under the alternative hypothesis. The mean of population 1 is unchanged.
S1 and S2 are the population standard deviations. They represent the variability in the populations.

Summary Statements

Group sample sizes of 24 and 24 achieve 81% power to detect a difference of 22.2 between the null hypothesis that both group means are 316.5 and the alternative hypothesis that the mean of group 2 is 294.3 with known group standard deviations of 25.9 and 25.9 and with a significance level (alpha) of 0.05000 using a two-sided Mann-Whitney test assuming that the actual distribution is normal.

5) Recruitment plan for study participants

Patients who are scheduled for a cesarean section under spinal anesthesia as an elective surgery at Chung-Ang University Hospital and who consent to participate in this study will be selected as study participants. No separate recruitment notice is necessary.

10. Methods

1) Study methods

Among patients scheduled for Cesarean section under spinal anesthesia, those who agreed to participate in this study will be randomly assigned to a control group (group C) and a palonosetron group (group P) according to a random allocation table.

The researcher in charge of group allocation will prepare the medication according to the assigned group and place it in an envelope with only the subject's number written on it. The envelope will be handed over to the researcher in charge of anesthesia, who will not be involved in any other stages of the study (group C: ondansetron 4mg/2ml, group P: palonosetron 0.075mg/1.5ml mixed with saline 0.5ml to a total of 2ml).

Spinal anesthesia will be performed by an anesthesiologist specializing in anesthesia and pain medicine who will not be involved in other stages of the study. The medication corresponding to each group will be administered intravenously 10 minutes before spinal anesthesia. Blood pressure will be measured at 3-minute intervals using a non-invasive blood pressure monitor, and the mean value will be recorded as the baseline value.

After spinal anesthesia, blood pressure will be measured at 1-minute intervals until delivery of the fetus. Phenylephrine infusion will be started at a rate of 0.24mcg/kg/min, and if the blood pressure falls below 80% of the baseline value, a phenylephrine bolus of 50mcg will be administered. If the blood pressure exceeds 120% of the baseline value, the infusion will be stopped.

If the heart rate falls below 55 beats/min, 0.5mg of atropine will be administered. The total amount of phenylephrine used until delivery of the fetus will be recorded, and the 1-minute and 5-minute Apgar scores will be recorded as neonatal outcomes.

2) Control group setting and randomization method

1:1 random assignment according to the randomization table

3) Study drugs

Palonosetrron group: palonosetron 0.075mg (1.5ml) and saline 0.5ml IV administration.
Control group: ondansetron 4mg/2ml IV administration.

4) Outcomes

- Primary outcome
  - Total amount of phenylephrine used up to the time of fetal delivery
- Secondary outomes
  - The lowest SBP, DBP, MBP, HR during the observation period
  - Presence or absence of hypotesion
  - Presence of Bradycardia
  - Presence of Nausea/vomiting
  - Whether sheivering occurs기타 관찰 항목
  - Age, Sex, Weight, Height, BMI, ASA grade, medical history, gestational age
  - Sensory block level corresponding to the 30th minute after spinal anesthesia
- Observation method

- Non-invasive monitoring for measuring the vital sign.
- For the total usage of phenylephrine, record the total usage recorded on the infusion pump at the time of delivery of the fetus and bolus usage.

5) Efficacy evaluation criteria, evaluation method

Compare the total amount of phenylephrine used between the two groups.

6) Differences from existing treatments or studies

Previous studies have reported that ondansetron and ramosetron, which are used as antiemetics, reduce hypotension caused by spinal anesthesia. However, research on palonosetron, a next-generation drug, has not yet been reported. In addition, when it is considered that ondansetron and ramosetron are preferably administered 30 minutes before the end of surgery for the purpose of preventing PONV, there is a problem that the effect of reducing PONV may be reduced. Considering that palonosetron is administered for the purpose of preventing PONV right before induction of anesthesia, if the drug reduces hypotension caused by spinal anesthesia, it is more ideal because it can expect two effects.

7) Benefits and risks of subjects

Not only can the use of palonosetron prevent PONV, but if the occurrence of hypotension after spinal anesthesia is reduced by using palonosetron, safer anesthesia management can be achieved, which will help fetal well-being.
There are no other risk factors because palonosetron is used at doses that are generally safely used.

8) Criteria for dropping out

- Those who refused to participate in the study.
- In the case of unexpected situations such as changes in the anesthesia plan due to incomplete spinal anesthesia.

9) Safety evaluation criteria, evaluation methods and reporting methods including side effects

- Reported side effects associated with the use of Palonosetron
- Treatment is performed for side effects that occur, and if they are harmful to the patient's health, the responsible researcher reports them to the IRB of Chung-Ang University Hospital.
-

10) Data Safety Monitoring Plan (DSMP)

As the data safety officer, the principal investigator will collect and review study data once a month, manage and monitor continuously for safety, and ensure the integrity of the data and the stability of the study subjects.

11) Data analysis and statistical analysis methods

For continuous data, the Shapiro-Wilk test will be used to confirm normality of the collected data. If normal distribution is achieved, the t-test will be used, otherwise, the Mann-Whitney U test will be used. For discontinuous data, chi-squared analysis or Fischer’s exact test will be used. A p-value of less than 0.05 is considered statistically significant. Statistical analysis will be performed using SPSS 15.0, and normally distributed data will be presented as mean ± standard deviation, while data that do not have a normal distribution will be presented as median (interquartile range).

12) Schedule of study implementation

- IRB approval date to 9 months: data collection
- 9 months to 1 year: data analysis and paper writing

11. Measures to protect the safety of subjects

1) Basic plan for ensuring research ethics

This clinical study will be conducted after being reviewed by IRB of the Central University Hospital. The information of the subjects participating in this study will only be used for the purpose of the study by medical staff who participate in the clinical research. Even if the results of the clinical trial are published in the future, the identity of the research subjects will never be exposed.

2) Informed consent process

Consent will be obtained directly from the patient through the principal investigator or co-researcher. The explanation and consent acquisition will be carried out without coercion, the day before the surgery, and there will be no unfair influence due to non-participation in the study. The explanation will be given in a way that the patient can easily understand, and the consent form will be signed directly by the patient.

3) Compensation plan

If physical damage occurs to the subjects due to participation in the clinical trial, appropriate treatment or treatment opportunities will be provided through the clinical trial responsibility (person in charge) before monetary compensation is confirmed.

4) Personal information protection plan

The personal information of research subjects will be stored in an encrypted file that only the principal investigator and co-researchers can access, and will be kept for approximately 3 years after the end of the research before being destroyed.

5) In case of including vulnerable research subjects

None

12. The storage and disposal methods for human-derived materials.

None

13. References

1) Chattopadhyay S, Goswami S. Palonosetron Versus Ramosetron Prophylaxis for Control of Postoperative Nausea and Vomiting after Cesarean Delivery under Spinal Anesthesia. Journal of obstetrics and gynaecology of India. 2015;65(1):28-33.

2) Shin HJ, Choi ES, Lee GW, Do SH. Effects of Preoperative Serotonin-Receptor-Antagonist Administration in Spinal Anesthesia-Induced Hypotension: A Randomized, Double-blind Comparison Study of Ramosetron and Ondansetron. Regional anesthesia and pain medicine. 2015;40(5):583-8.

3) Varshney RK, Garg M, Kapoor K, Jheetay GS. The role of ramosetron in the prevention of post-spinal shivering in obstetric patients. A prospective randomized double blind study. Romanian journal of anaesthesia and intensive care. 2019;26(1):37-43.

4) Xiao F, Wei C, Chang X, Zhang Y, Xue L, Shen H, et al. A Prospective, Randomized, Double-Blinded Study of the Effect of Intravenous Ondansetron on theEffective Dose in 50% of Subjects of Prophylactic Phenylephrine Infusions for Preventing Spinal Anesthesia-Induced Hypotension During Cesarean Delivery. Anesthesia and analgesia. 2020;131(2):564-9.
